# The choice of biopolymer is crucial to trigger angiogenesis with vascular endothelial growth factor releasing coatings

**DOI:** 10.1007/s10856-020-06424-3

**Published:** 2020-10-27

**Authors:** Christiane Claaßen, Miriam Dannecker, Jana Grübel, Maria-Elli Kotzampasi, Günter E. M. Tovar, Boris V. Stanzel, Kirsten Borchers

**Affiliations:** 1 Institute of Interfacial Process Engineering and Plasma Technology IGVP, University of Stuttgart, Nobelstr. 12, 70569 Stuttgart, Germany; 2 Fraunhofer Institute for Interfacial Engineering and Biotechnology IGB, Nobelstr. 12, 70569 Stuttgart, Germany; 3 Department of Ophthalmology, University of Bonn, Ernst-Abbe-Str. 2, 53127 Bonn, Germany; 4 Augenklinik Sulzbach, Knappschaftsklinikum Saar, An der Klinik 10, 66280 Sulzbach, Germany; 5 Fraunhofer Institute for Biomedical Engineering IBMT, Joseph-von-Fraunhofer-Weg 1, 66280 Sulzbach/Saar, Germany

## Abstract

Bio-based coatings and release systems for pro-angiogenic growth factors are of interest to overcome insufficient vascularization and bio-integration of implants. This study compares different biopolymer-based coatings on polyethylene terephthalate (PET) membranes in terms of coating homogeneity and stability, coating thickness in the swollen state, endothelial cell adhesion, vascular endothelial growth factor (VEGF) release and pro-angiogenic properties. Coatings consisted of carbodiimide cross-linked gelatin type A (GelA), type B (GelB) or albumin (Alb), and heparin (Hep), or they consisted of radically cross-linked gelatin methacryloyl-acetyl (GM5A5) and heparin methacrylate (HepM5). We prepared films with thicknesses of 8–10 µm and found that all coatings were homogeneous after washing. All gelatin-based coatings enhanced the adhesion of primary human endothelial cells compared to the uncoated membrane. The VEGF release was tunable with the loading concentration and dependent on the isoelectric points and hydrophilicities of the biopolymers used for coating: GelA-Hep showed the highest releases, while releases were indistinguishable for GelB-Hep and Alb-Hep, and lowest for GM5A5-HepM5. Interestingly, not only the amount of VEGF released from the coatings determined whether angiogenesis was induced, but a combination of VEGF release, metabolic activity and adhesion of endothelial cells. VEGF releasing GelA-Hep and GelB-Hep coatings induced angiogenesis in a chorioallantoic membrane assay, so that these coatings should be considered for further in vivo testing.

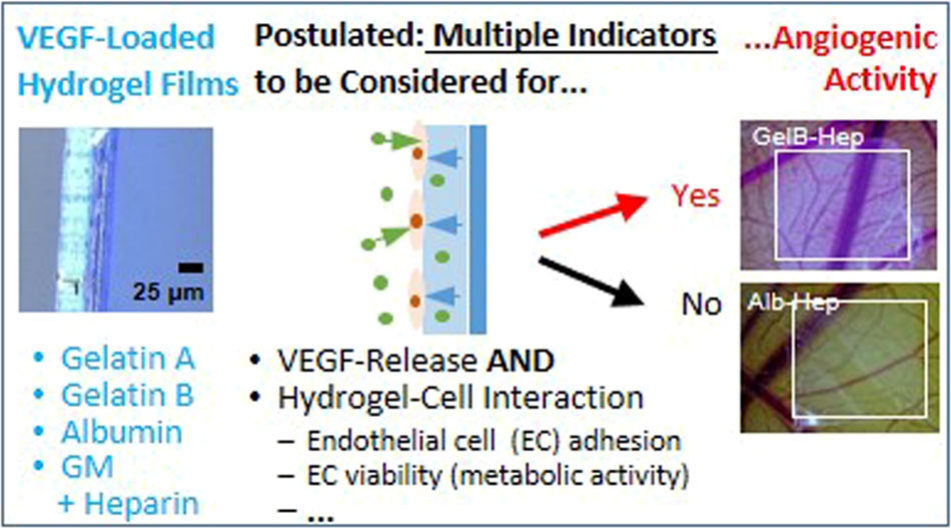

## Introduction

Presently, formation of fibrotic encapsulation around implants and tissue engineered grafts remains a fundamental limitation in clinical translation [1, 2]. Biocompatible hydrogel coatings are suggested to reduce the foreign body response to implants and thereby enhance their biocompatibility [1, 3]. Gelatin- and albumin-based materials have been widely studied as biopolymer-based coatings for polymeric implants: Gelatin coating of polymer substrates was, for example, investigated to enhance the biocompatibility of arterial prostheses and in artificial blood vessel engineering [4–7], or other cardiovascular applications [8, 9]. Albumin [10], combinations of albumin and heparin [11, 12] and also combinations of albumin and gelatin [7] were for example studied as surface coating to improve endothelialization and hemocompatibility of polymeric implants.

Long-term function of implants has been proposed to be improved by hydrogel coatings releasing drugs locally controlled, e.g., to prevent infection by releasing antibacterial proteins [13], or to reduce the formation of fibrotic encapsulations by releasing pro-angiogenic growth factors [14]. In particular, controlled release of pro-angiogenic growth factors from hydrogel coatings is expected to stimulate improved bio-integration of implants [10, 14, 15]. For this matter gelatin type B (GelB) coatings releasing basic fibroblast growth factor (bFGF) to induce angiogenesis were investigated in vivo so far [14, 15], and albumin coatings were for example loaded with vascular endothelial growth factor (VEGF) [10].

Gelatin is produced by partial hydrolysis of collagen, mainly type I, via an acidic hydrolysis leading to gelatin type A (GelA) with an isoelectric point (IEP) at approximately pH 9.0, or via an alkaline hydrolysis leading to GelB with an IEP at approximately pH 5.0 [16]. Albumin is mostly produced biotechnologically and has an IEP at pH 4.7 [17]. Consequently, GelA is positively charged at neutral pH, while GelB and albumin are negatively charged. The IEP of gelatin has been described to affect the loading and release behaviour of various growth factors [18–22] and attributed to the formation of polyion complexes of GelB and growth factors, due to opposite charging at neutral pH [23, 24].

In a recent study we showed that the IEP governed the release of VEGF from carbodiimide cross-linked GelA-heparin, and albumin-heparin as well [25]. In another study, we investigated radically cross-linkable gelatin methacryloyl(-acetyl) (GM/A) as storage and release system for VEGF and found that the release was controlled by the various interactions between growth factor and polymer, i.e. besides charging and affinity, the hydrophilicity of the hydrogels played a role as well [26].

With regard to improve the functionality of implant surrounding tissue, or even implanted artificial tissue which was cultured in vitro by stimulation of capillary ingrowth, it seems so far unclear from literature which material system would be most qualified. In the current study, we developed VEGF releasing coatings based on carbodiimide cross-linked GelA-heparin, GelB-heparin, albumin-heparin, and radically cross-linked methacryl-modified gelatin-heparin (GM5A5-HepM5) on polyethylene terephthalate (PET) substrates. PET substrates are for example of interest as cell carriers for ophthalmic applications [27–30]. We addressed in particular the immobilization stability, adhesion of endothelial cells, VEGF release, and the induction of angiogenesis in a chorioallantoic membrane (CAM) assay. With that, we aimed to contribute to the knowledge about cell adhesive, pro-angiogenic coatings for polymeric substrates and allocate a decision-making basis for which coating to investigate further in in vivo studies.

## Materials and methods

### Materials

Human recombinant albumin (HSA), 1-Ethyl-3-(3-dimethylaminopropyl)carbodiimide (EDC), fluoresceindiacetat (FDA), 2-(*N*-Morpholino)ethansulfonsäure (MES), *N*-Hydroxysuccinimide (NHS), propidium iodide (PI), acetic anhydride (AcAnh), methacrylic anhydride (MAAnh), sodium hydroxide (NaOH), ammonium peroxodisulfate (APS), *N*,*N*,*N*′,*N*′-Tetramethylethylenediamine (TEMED), 2,2′-Azino-bis(3-ethylbenzothiazoline-6-sulfonic acid) (ABTS), sulphuric acid, magnesium chloride (MgCl_2_), sodium acetate (AcNa), acetic acid (AcH), bovine serum albumin (BSA), sodium dodecyl sulfate (SDS) and Dulbecco’s phosphate buffered saline without (PBS^−^) or with MgCl_2_ and CaCl_2_ (PBS^+^) were purchased from Sigma Aldrich (Germany). Dispase, fetal calf serum (FCS), Gibco^®^ versene solution and trypsin were purchased from Thermo Fisher Scientific (Germany). Tween^®^-20 and sodium 3-trimethylsilyl-propionate-2,2,3,3-*d*4 (TMSP) were purchased from Merck (Germany). Other reagents were purchased from the following sources (given in parentheses): Alcian blue 8GX (Merck Millipore; Germany), glutaraldehyde solution (50%, Merck Millipore; Germany), Casyton (Omni Life Science, Germany), Cell Titer 96 Aqueous Solution Cell Proliferation Assay (Promega, Germany; including 3-(4,5-dimethylthiazol-2-yl)-5-(3-carboxymethoxyphenyl)-2-(4-sulfophenyl)-2H-tetrazolium (MTS)), Endothelial Cell Growth Medium MV SupplementPack (ECGM, PromoCell, Germany), deuterium Oxide (Deutero; Germany), penicillin/streptomycin (Thermo Fisher Scientific; Germany), heparin sodium salt (Celsus Laboratories; USA), gelatin type A (MedellaPro^®^, pig skin North America, bloom 233, Gelita; Germany), gelatin type B (Limed, bovine bone, 232 Bloom, Gelita; Germany), *rh*VEGF165 (VEGF, Morphoplant; Germany), human VEGF Standard ELISA development kit (PeproTech; Germany), PET membranes (pore size 1 µm, thickness 11 µm, diameter 25 mm; it4ip; Belgium), fertilized chicken eggs (Lohmann White, LSL Rhein-Main; Germany). Buffers required for the VEGF-ELISA were prepared with a 0.01 M PBS with pH 7.2, without magnesium and calcium ions (PBS^−^). Dialysis was conducted using dialysis membranes (MWCO 12 kDa–14 kDa for gelatin; MWCO 3.5 kDa for heparin) from Medicell International Ltd (UK).

### Preparation of carbodiimide cross-linked hydrogel thin films

#### Pre-activation of membrane

Each PET membrane was incubated in 2 mL NaOH (1% w/v) at 37 °C for 30 min under gentle shaking and afterwards washed in deionized water to obtain a clean surface. Then, the membranes were incubated in 2 mL EDC/NHS solution each (0.1 M EDC, and 5 mM NHS in PBS^+^) for 10 min at 37 °C under gentle shaking for pre-activation of surface carboxyl groups.

#### Film preparation and cross-linking

All stock solutions were prepared in PBS^+^ (pH = 7.3) for GelA and GelB, and in MES buffer (pH = 4.5) for albumin: 12.5% (w/w) gelatin/albumin, 10% (w/w) heparin, 2 M EDC. Heparin was pre-activated using 25 μL heparin stock solution, 18.75 μL EDC stock solution, and 6.25 μL PBS^+^ (for GelA and GelB)/MES (for albumin) for 10 min at room temperature (RT). Then 200 µL of gelatin/albumin stock solution were added. Coatings on the PET membrane were prepared by doctor blading on a glass plate, directly after adding the gelatin/albumin to the heparin solution: In case of gelatin doctor blade and plate were pre-heated. 200 µL of the hydrogel precursor solution were pipetted on each pre-activated, dried PET membrane and distributed using the 25 µm side of the doctor blade. The films were left for cross-linking overnight at 4 °C or for 2 h (gelatin)/4 h (albumin) at 37 °C in a humidified atmosphere, then washed in PBS^+^ at 37 °C for 24 h under gentle shaking and subsequently dried under vacuum for 1 h at 60 °C. Prior to use, membranes were re-swollen in release medium (70 µg BSA/mL in PBS^+^ + 1% penicillin/streptomycin) at 37 °C under gentle shaking overnight unless specified otherwise for further experiments.

### Preparation of radically cross-linked hydrogel thin films

#### Synthesis of GM

Gelatin functionalized with methacrylic (GM5) or methacrylic and acetic residues (GM5A5) was prepared and characterized according to a previously described procedure from gelatin type A [31]. The degree of methacryloylation was determined by NMR spectroscopy using the TMSP-method [31] to be 0.618 ± 0.032 mmol/g (GM5; *n* = 3) and 0.638 ± 0.023 mmol/g (GM5A5; *n* = 3); the total degree of modification (methacryl + acetyl amount) for GM5A5 was determined to be 0.807 ± 0.162 mmol/g (*n* = 3).

#### Synthesis of HepM

Heparin methacrylate (HepM5) was prepared and characterized according to a previously described procedure [26]. The degree of methacryloylation was determined by NMR spectroscopy using the TMSP-method [31] to be 0.171 ± 0.003 mmol/g (*n* = 3).

#### Pre-coating of membrane

Changed according to a procedure published in [32], a solution of 6% glutaraldehyde and 12% GM5 (1:1) in H_2_O was prepared and the pH was adjusted to 5.2 with 0.1 M sulfuric acid. Each PET membrane was incubated in 2 mL NaOH (1% w/v) at 37 °C for 30 min under gentle shaking and afterwards washed in deionized water to obtain a clean surface. Then, the membranes were incubated in the glutaraldehyde-GM5 solution (2 mL per membrane) for 2 h at room temperature. Before subsequent film deposition, the activated membranes were washed with distilled water.

#### Film preparation and cross-linking

Stock solutions were prepared in PBS^+^ (pH = 7.3) for GM5A5 (20% w/w), HepM5 (10% w/w), APS (2 M), TEMED (0.5 M). Stock solutions were mixed in a hydrogel precursor solution to the final concentrations of 10% GM5A5, 1% HepM5, 0.075 M APS and 0.0188 M TEMED. The pre-coated membranes were placed in aluminium moulds (27 mm diameter) with a 50 µm deep recess, the hydrogel precursor solution was pipetted into the mould and the mould was covered with a quartz glass pane simultaneously. Hydrogel coatings were left for radical cross-linking 2 h at 37 °C. Subsequently the glass pane was removed and the coated membranes were then washed in PBS^+^ at 37 °C for 24 h under gentle shaking and subsequently dried under vacuum for 1 h at 60 °C. Prior to use for further experiments membranes were re-swollen in release medium at 37 °C under gentle shaking overnight unless specified otherwise.

### Surface characterization of coated and uncoated PET membranes

Unless specified otherwise, coated membranes were used before re-swelling as described in 2.2 and 2.3, uncoated membranes were treated with NaOH as described in 2.2 and 2.3, respectively, and used after drying under vacuum for 1 h at 60 °C.

Membranes were characterized using scanning electron microscopy (SEM), X-ray photoelectron spectroscopy (XPS), infrared spectroscopy (IR) and contact angle measurements. SEM pictures of dry membranes were collected on a Zeiss Leo 1530 VP (Jena, Germany) to analyze the surface morphology. IR spectra were collected on a Vertex 70 spectrometer (Bruker, Germany) (spectra see supporting information). Water-air contact angles of membranes re-swollen in ultrapure water were measured using the captive bubble method with an OCA40 (Dataphysics, Germany). XPS spectra were measured on an AXIS Supra surface analysis instrument (Kratos Analytical, England).

### Determination of film thickness and long-term stability

Film thickness and long-term stability of hydrogel coatings were determined after re-swelling (24 h), and after 7 d, 14 d and 28 d of incubation in release medium at 37 °C under cell culture conditions. Membranes were stained by alcian blue staining (gelatin/albumin-coatings: 0.05% alcian blue in 0.025 M acetate buffer (pH = 5.8) with addition of 0.3 M MgCl_2_; GM5A5-coatings: 1% alcian blue in 3% acetic acid (pH ≈ 2.5)) for 24 h or 1 h (GM5A5-HepM5-coatings) at RT under gentle shaking in the dark. Membranes were then washed with deionized water, photographed and cut into quarters using a scalpel. From each quarter a thin stripe was cut and placed with the cut edge facing upwards on a double-sided tape in a petri dish. A drop of water was put on the strip for 1 min to ensure full re-swelling. Light microscopy of the layer and thickness determination was done at ×500 magnification.

### Loading and release of vascular endothelial growth factor

For the determination of the release properties for VEGF, coated and re-swollen membranes were prepared according to 2.2 and 2.3. They were loaded with VEGF by using 2 mL VEGF solution per membrane (0.1 µg VEGF per mL or 1.0 µg VEGF per mL in PBS^+^ + 70 µg BSA/mL + 1% penicillin/streptomycin) and incubated for 1 h at 37 °C under gentle shaking. After the loading time the VEGF solution was replaced by 2 mL release medium. The release medium was changed at predetermined time points (0.25 d, 1 d, 2 d, 5 d, 7 d, 14 d, 21 d and 28 d) and was stored at −19 °C until analysis for typically maximum 4 weeks. Quantification of VEGF content in the samples was performed using an ELISA-kit following the manufacturer’s instructions. The absorption was measured using a fluorescence microwell plate reader, Tecan Synergy2 Multi-Mode Microplate Reader, from BioTek (Germany) for the last step of the ELISA protocol.

### Cell adhesion

Endothelial cells were isolated from human skin biopsies received from the Robert-Bosch-Krankenhaus, Klinik Charlottenhaus, Stuttgart (Germany) with given consent of each donor and cultivated as previously described in [33]. Hydrogel-coated PET membranes were prepared as described in 2.2 and 2.3, uncoated PET membranes, polystyrene (PS) and tissue culture polystyrene (TCPS) served as controls for all experiments. The re-swollen membranes were placed in 6-well TCPS-plates; Teflon rings were put on top to prevent floating of the membranes. 3 mL ECGM were added per well and membranes were incubated overnight at 37 °C, 5% CO_2_ and 95% humidity. Then the medium was aspirated and 2 mL of a endothelial cell suspension (passage 3) with 36,075 cells/mL medium (=7500 cells per cm²) were pipetted onto the membranes and cell adhesion was allowed for 24 h under standard cell culture conditions (37 °C, 5% CO_2_ and 95% humidity). Then rings were removed and the membranes were washed with PBS^+^ to remove non-adherent cells. Adherent cells were stained through live-dead-staining (1960 μL PBS^+^, 20 μL PI-solution and 20 μL FDA-solution) for 3 min in an incubator and washed with PBS^+^. Fluorescence microscopy was done at ×2, ×4, and ×10 magnification. Disturbing background signals were reduced by black balance and haziness was corrected with haze reduction. Numbers of livings cells were counted from ×2 magnification and overlay-pictures were prepared in the ×4 and ×10 magnification.

### Chorioallantoic membrane assay

#### Incubation of the eggs

Fertilized chicken eggs were pre-incubated for 72 h at 37.5 °C with 61% humidity and turned every 2 h. The ex-ovo method was used and incubation devices were prepared as described before in [34]. Under sterile conditions the egg shells were cracked on a sharp edge of a bowl, the egg content was transferred carefully into the incubation device and the incubation device was placed in an incubator (37.5 °C, 70% humidity and 2% CO_2_), and incubated for another 120 h. The embryos were controlled every day and dead embryos were removed from the incubator.

#### Sample application

On embryonic day 9 (counted from the beginning of the pre-incubation) test materials were placed on the CAM of the chicken embryos in order to evaluate if the released VEGF induced a pro-angiogenic response. Re-swollen hydrogel-coated PET-membranes were cut into squares of 5 mm × 5 mm and loaded with VEGF by incubation in growth factor solution (50 µg VEGF per mL in release medium; 100 µL per sample) for 1 h at 37 °C. Coated membranes incubated in release medium without addition of growth factor served as control. Six samples were applied on each embryo and were placed on the CAM distant from main blood vessels. Photographs were taken every 24 h to control the development of the embryos and the capillary system of the CAM.

#### Evaluation of a pro-angiogenic effect

On embryonic day 12 the embryos were transferred in a petri dish and the samples with the surrounding capillary system were observed with a digital microscope. Afterwards the embryos were sacrificed by decapitation.

### Statistical analysis

Statistical analysis was performed using Student’s *t* test. *P* values less than 0.05 were considered statistically significant. All data are presented as mean ± standard deviation. Unless stated otherwise, the value of *n* is defined as the number of independently performed experimental iterations.

## Results

### Surface characterization of coated and uncoated PET-membranes

**Fig. 1:**
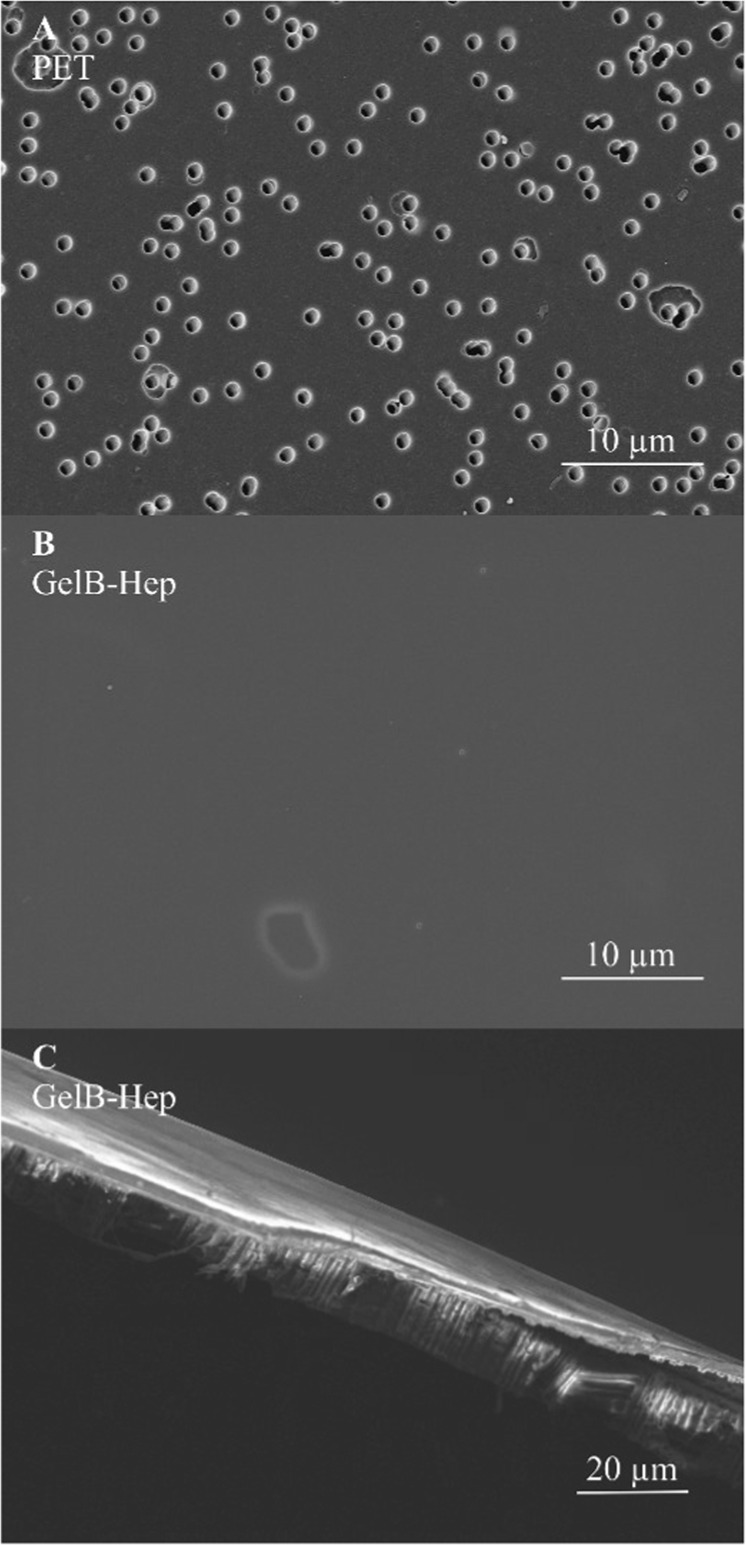
Representative scanning electron microscopy images of the uncoated polyethylene terephthalate (PET) membrane (**a**), the top side of a GelB-Hep coated membrane (**b**) and a cross-section of a GelB-Hep coated membrane (**c**)

The uncoated membranes were characterized via XPS, SEM and contact angle measurements to examine surface chemistry and structure. SEM measurements showed a homogeneous distribution of pores with a size of approx. 1 µm as expected from the manufacturer’s specifications (Fig. 1a). According to XPS analysis, the membranes had carboxyl and hydroxyl groups on the surface from the track-etching process (supporting information Table S1).

Four different types of hydrogel coatings were investigated in this study with regard to their suitability as cell-adhesive, pro-angiogenic coatings for PET. Hydrogel coatings consisted of 10% protein (gelatin type A, gelatin type B, or human serum albumin) and 1% heparin, and were cross-linked via carbodiimide chemistry, referred to as GelA-Hep, GelB-Hep and Alb-Hep. Alternatively, the hydrogel coatings consisted of 10% gelatin methacryloyl-acetyl and 1% heparin methacrylate which were cross-linked radically, referred to as GM5A5-HepM5. The carbodiimide cross-linked coatings were applied to the PET membrane via doctor blading and immobilized by pre-activation of the membranes’ carboxyl groups with carbodiimide. The radically cross-linked coating was applied to the PET membrane in an aluminum mould and covered with glass during cross-linking. Cross-linking of GM5A5-HepM5 films was not successful if atmospheric oxygen interfered with the radical reaction (data not shown); therefore, simple doctor blading was not possible. Immobilization of GM5A5-HepM5 was achieved by pre-treatment of the membrane with glutaraldehyde and pre-coating with GM5.

The protein layers formed smooth coatings at the membrane surface as can be recognized from exemplary SEM pictures (Fig. 1b). Representative cross-sections showed thicknesses of the dry coating between 1.6 and 2.5 µm (Fig. 1c).

IR-ATR spectra of the coated membranes showed bands from the biopolymer coatings but not from the underlying PET membranes (see Supporting Information Fig. S1). Spectra were almost identical for all gelatin-based coatings. Similar bands but differences in the finger print region occurred for the albumin-heparin coating.

The water contact angles of the GelA-Hep and GelB-Hep coated PET (Table 1) were smaller than of uncoated PET (*p* < 0.05), while Alb-Hep and GM5A5- HepM5 coatings did not change the contact angle significantly (*p* > 0.2).

**Table 1:** Water contact angles of the uncoated polyethylene terephthalate membrane (PET) and the biopolymer coated membranes

Material	Water contact angle in °
PET	43.2 ± 2.2
Alb-Hep	44.5 ± 2.2
GelA-Hep	39.5 ± 1.9
GelB-Hep	35.8 ± 3.3
GM5A5-HepM5	41.7 ± 4.1

Contact angles were determined using the captive bubble method. (*n* = 3)

### Characterization of swollen hydrogel thin films on PET membrane

**Fig. 2:**
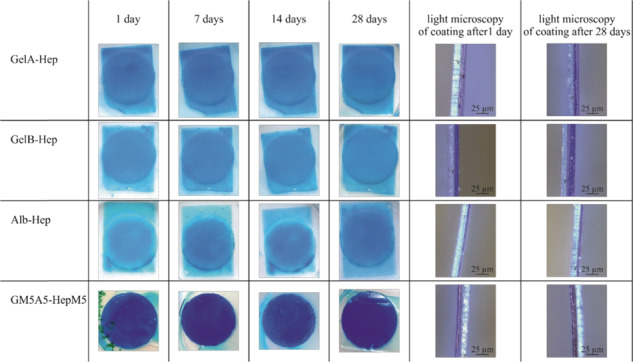
Alcian blue staining of biopolymer-coated polyethylene terephthalate membranes after 1 d, 7 d, 14 d and 28 d of incubation in release medium at 37 °C under cell culture conditions (left) and representative light microscopic images of the respective swollen coating after 1 d and 28 d (right). All coatings show a homogeneous staining intensity indicating homogeneous coatings and no coating detachment or inhomogeneity after the incubation (*n* = 3)

**Fig. 3:**
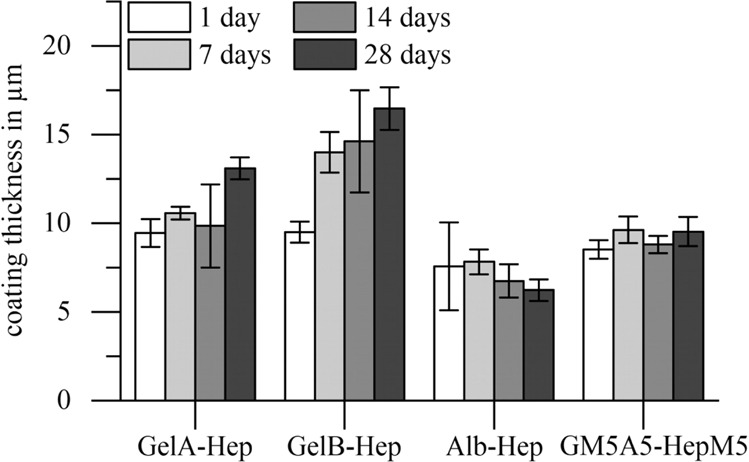
Film thicknesses of biopolymer coatings on polyethylene terephthalate (PET) membranes directly after washing (1 day) and after incubation in release medium at 37 °C for 7 days, 14 days and 28 days. Film thickness of GelA-Hep and GelB-Hep coatings increased significantly during the incubation (*p* < 0.01), while thicknesses of Alb-Hep and GM5A5-Hep remained almost constant (*p* > 0.05) (*n* = 3)

The homogeneity, thickness and stability of swollen hydrogel coatings were characterized applying alcian blue staining and light microscopy-based measurement of the film thicknesses (Figs. 2 and 3). The use of alcian blue staining was described by our group in the context of proofing heparin-functionalization of hydrogels [25, 26]. In the present study, the staining was applied to visualize the coatings and assess their homogeneity.

All hydrogel coatings showed a homogeneous blue staining after re-swelling of the coated and washed membranes (1 day, Fig. 2, left). No visible coating detachments or inhomogeneities after the incubation in release medium for max. 28 days at 37 °C were observed (Fig. 2, left). The coating thicknesses were determined in the swollen state by cutting thin stripes of the membranes and evaluating the coating thickness via light microscopy (Fig. 2, right). Measurements were performed at more than 16 positions on the membranes.

The thicknesses of the different biopolymer coatings (Fig. 3) did not differ significantly initially after re-swelling (*p* > 0.05). However, the film thickness of GelA-Hep and GelB-Hep coatings increased during the 28 days of incubation. GelA-Hep was 9.5 ± 0.8 µm thick after re-swelling and significantly thicker with 13.1 ± 0.6 µm (+39%) after 28 days incubation in buffer solution (*p* < 0.01). GelB-Hep showed the highest increase in thickness with 9.5 ± 0.6 µm after re-swelling and 16.5 ± 1.2 µm (+73%) after 28 days of incubation (*p* < 0.01). Alb-Hep showed a slight decrease in thickness; coatings were 7.6 ± 2.5 µm after re-swelling and 6.2 ± 0.6 µm (−12%) after incubation for 28 days (*p* > 0.2). GM5A5-HepM5 showed a slight increase in thickness with 8.5 ± 0.5 µm after re-swelling and 9.5 ± 0.8 µm (+12%) after incubation for 28 days (*p* > 0.05).

### Assessment of endothelial cell adhesion

The cytocompatibility of the coating materials and its capability of supporting endothelial cells were investigated with primary HDMVEC. Acute cytotoxic effects of the materials can be precluded on the base of extract testing. HDMVEC showed increased metabolic activity in Gel-Hep extracts (*p* < 0.05) and similar viabilities in Alb-Hep (*p* > 0.1) or GM5A5-HepM5 (*p* > 0.1) extracts compared with the negative control (see Supporting Information Fig. S2).

**Fig. 4:**
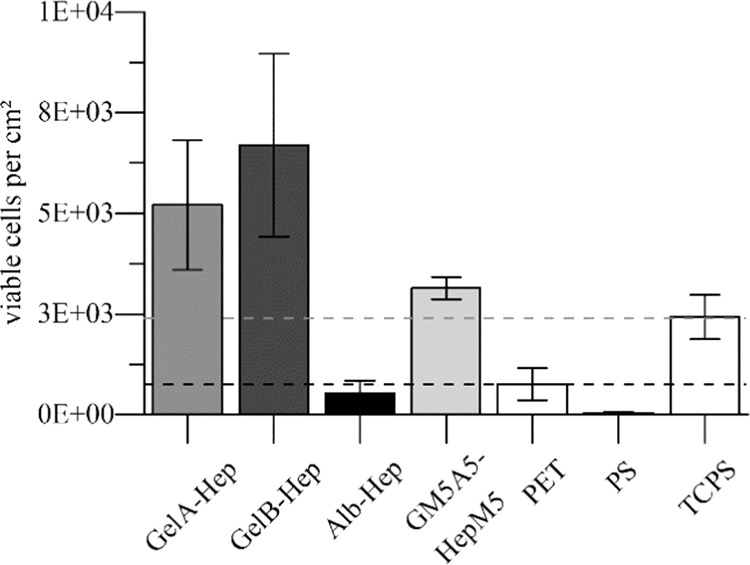
Cell adhesion to hydrogel coated polyethylene terephthalate membranes (GelA-Hep, GelB-Hep, Alb-Hep, GM5A5-HepM5), uncoated membranes (PET), polystyrene (PS) and tissue culture polystyrene (TCPS). All gelatin-based coatings increased cell adhesion compared to PET (*p* < 0.001), and showed even higher cell numbers than TCPS (*p* < 0.05) (*n* ≥ 7 for GelA-Hep, GelB-Hep, PET and TCPS; *n* ≥ 3 for GM5A5-Hep, Alb-Hep and PS)

Endothelial cell adhesion to the coatings was investigated using live-dead staining after 24 h. The adherent, viable cells of at least three different donors were counted, and cell numbers were compared between the coated membranes, uncoated PET membranes, PS and TCPS. Representative images of the live-dead staining for the different materials can be found in the supporting information. Endothelial cell adhesion was on all surfaces higher than on PS (*p* < 0.001). The numbers in Fig. 4 show that endothelial cell adhesion to PET and Alb-Hep coated PET were similar (*p* > 0.1) and significantly lower compared to TCPS (*p* < 0.001). All gelatin-based coatings enhanced the endothelial cell adhesion compared to the uncoated PET membrane (*p* < 0.001) and showed significantly higher cell numbers compared to TCPS (*p* < 0.05). Cell adhesion between GelA-Hep and GelB-Hep was not significantly different (*p* > 0.1), while GM5A5-HepM5 showed lower cell numbers than GelA-Hep and GelB-Hep (*p* < 0.05). In relative numbers approximately 89% of the applied cells adhered to GelB-Hep, 69% to GelA-Hep, 42% to GM5A5-HepM5, 32% to TCPS, 10% to PET and 7% to Alb-Hep.

### Release of vascular endothelial growth factor from hydrogel coatings

The above mentioned hydrogel coatings (GelA-Hep, GelB-Hep, Alb-Hep, GM5A5-HepM5) were loaded with vascular endothelial growth factor (VEGF) by incubation in growth factor solution (2 mL per membrane with 0.1 µg or 1 µg VEGF per mL; incubation for 1 h at 37 °C under gentle shaking) in order to investigate the influence of the polymer nature and loading concentration onto the release of VEGF. Release experiments were conducted over 28 days at 37 °C under cell culture conditions in release medium (PBS^+^ + 70 µg/mL BSA) (all values for the release of VEGF can be found in the Supporting Information Table S2).

**Fig. 5:**
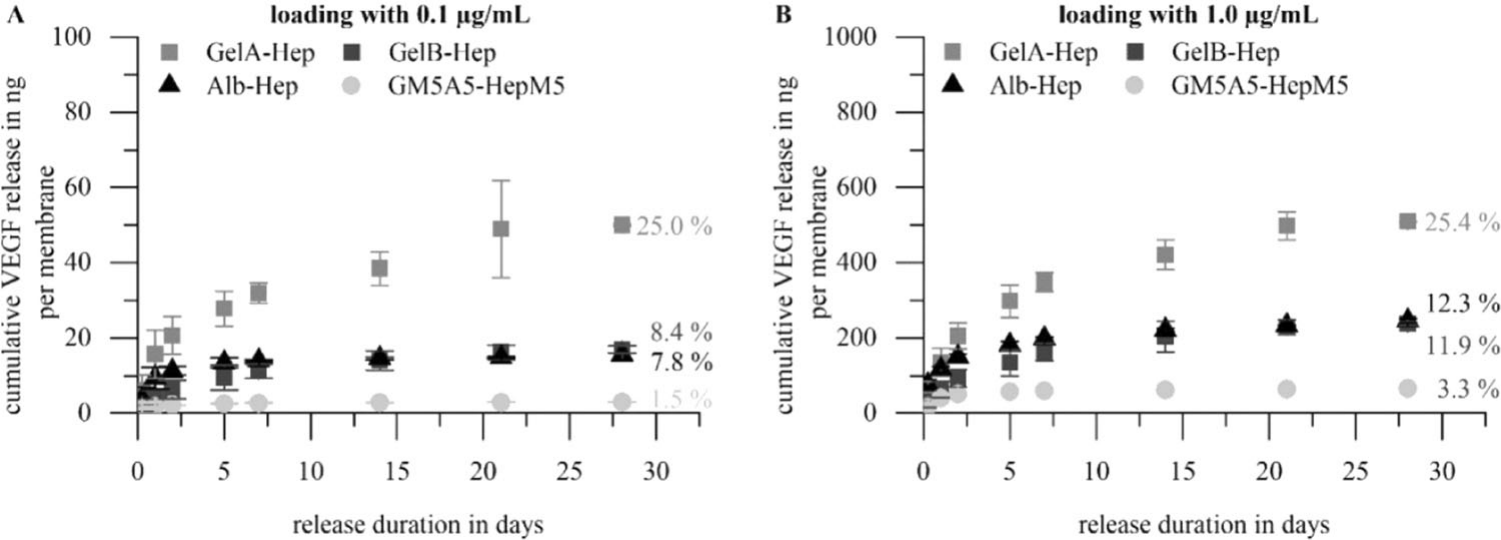
Release of vascular endothelial growth factor (VEGF) from biopolymer-coated polyethylene terephthalate (PET) membranes over 28 d at 37 °C in release medium. **a** Loading with 2 mL VEGF solution (0.1 µg VEGF per mL in release medium) per membrane; **b** Loading with 2 mL VEGF solution (1.0 µg VEGF per mL in release medium) per membrane. GelA-Hep coatings showed the highest release followed by GelB-Hep and Alb-Hep with similar releases, the smallest release was determined for the GM5A5-HepM5 coatings. (*n* = 3 for GM5A5-Hep and Alb-Hep; *n* = 6 for GelA-Hep and GelB-Hep). Percentage values were calculated based on the amount of VEGF applied for loading of the membranes

The absolute release from all hydrogel formulations was higher when loading was done with 2.0 µg VEGF per membrane compared to 0.2 µg (*p* < 0.01, Fig. 5a, b). The long-term release rates (day 7-day 28) were significantly higher for all hydrogel coatings loaded with 2.0 µg VEGF per membrane compared to the release rate for the same coating loaded with 0.2 µg (*p* < 0.01) as well. Concerning the percentage release of the coatings, these values were calculated based on the amount of VEGF applied for loading. Here, gelatin type A coated membranes released a similar percentage of VEGF at both loading concentrations overall, while the released percentages for the three other coatings had a trend to be lower at the lower loading concentration (Fig. 5a, b). In general, GelA-Hep coatings showed the highest release, releases from GelB-Hep and Alb-Hep were comparable and GM5A5-HepM5 coatings released the lowest amount of VEGF.

### CAM-assay

**Fig. 6:**
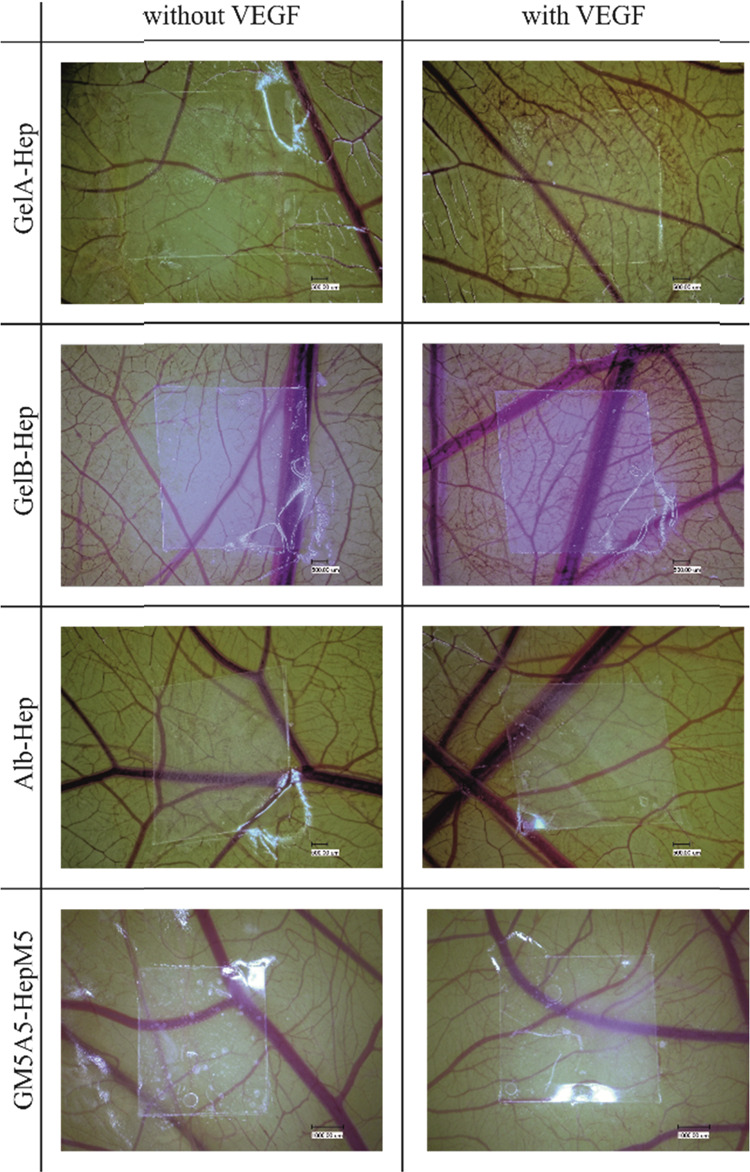
Chorioallantoic membrane assay of hydrogel coated polyethylene terephthalate (PET) membrane samples (GelA-Hep, GelB-Hep, Alb-Hep, GM5A5-HepM5), without loading of growth factor (left row) or loaded in a vascular endothelial growth factor solution (50 µg/mL VEGF in release medium) (right row) (representative photographs; *n* = 5 for GelA-Hep, GelB-Hep and GM5A5-HepM5; *n* = 3 for Alb-Hep)

The ability of the coatings to induce angiogenesis was investigated with an *ex ovo* CAM assay using hydrogel-coated membrane pieces of approx. 5 × 5 mm with or without loaded VEGF. The vascular structure under and in direct neighborhood of coated membrane samples was evaluated microscopically on embryonal day 12. Representative microscopic images of the different coatings are shown in Fig. 6.

VEGF loaded GelA-Hep and GelB-Hep coatings obviously induced formation of additional vessel structures, while the GelA-Hep and GelB-Hep coated membranes without VEGF loading showed no pro- or anti-angiogenic response.

In contrast, GM5A5-HepM5 or Alb-Hep coatings did not trigger any detectable response whether they were loaded with VEGF or not.

## Discussion

The carboxyl and hydroxyl groups that were detected via XPS on the surface of track-etched PET membranes were formed during the track-etching process and the treatment with sodium hydroxide solution. Carboxyl and hydroxyl groups were subsequently taken as anchor for the immobilization concept of the coatings: pre-incubation with the carbodiimide EDC activated carboxyl groups present at the membrane surface, which then reacted with free amino functions of the applied gelatin or albumin molecules. This simple method of surface functionalization of PET membranes is very much straightforward. Alternative solutions for immobilization of gelatin-based hydrogel coatings on PET were also suggested before e.g. the use of a perfluoroarylazide-functionalized *N*-hydroxy-succinimide to activate the membrane surface for subsequent protein coupling [35].

The specific difficulty in immobilization of highly modified GMA is the lack of amino functions in such derivatives. Therefore, the above-mentioned technique was not suitable for GM5A5-HepM5 coatings. Here, a two-step immobilization by use of less modified GM5 prove to be successful. We attribute the coupling of GM5 and the PET surface to the reactivity of glutaraldehyde with hydroxyl functions [36], which can be expected to be present in GM5 [31]. Cross-linking of GM5 and GM5A5 was achieved via radical chain-reaction of the methacrylic functions.

The showing of typical amide associated bands as the amine N–H stretch and C=O stretch within the IR spectra (see Supporting Information) of all coatings match with the expectations. The strongly pronounced signal at approx. 3288 cm^−1^ might be caused by additional secondary amines from the heparin molecules present within the coatings.

The resulting reduced contact angles for the coatings with unmodified gelatin are in accordance with literature [4, 8]. Thereby, the contact angle data give a first impression of the success of the coating process. Contact angles of albumin- or GM5A5-based PET coating have to our knowledge not been investigated so far. As albumin naturally possesses hydrophobic regions, and the integration of methacryl and acetyl residues added hydrophobic groups to the gelatin molecules, the slightly higher contact angles compared to Gel-Hep coatings seem consistent.

Looking at the wet film thickness, we assign the increase in film thickness of Gel-Hep gels to enhanced swelling capacity of the coatings due to hydrolytic break of biopolymer chains without substantial mass loss, while the downward trend in thickness of Alb-Hep gels might indicate actual loss of biopolymer because of more advanced degradation within the polymer layer. This interpretation is supported by earlier results obtained for bulk hydrogels of comparable composition [25]: There, no change in the equilibrium degree of swelling of GelA-Hep hydrogels was observed; but a significant increase in swelling capacity but without mass loss was found for Alb-Hep (incubation in buffer solution, 37 °C, 21 days). The accelerated hydrolysis occurring in the hydrogel thin films of the current study can be assigned to the higher surface to volume ratio, i.e. stronger proceeding of degradation in all gel types: degradation in Gel-Hep gels now led to an increase in swelling, and even stronger degradation in Alb-Hep coatings resulted in beginning biopolymer loss. The bonding of all hydrogels to the underlying PET was proved to be stable as no detachment was found even after 4 weeks of incubation. Altogether, the coatings showed good homogeneities and stabilities even after 4 weeks of incubation under cell culture conditions.

Comparing the data obtained in this study for increased endothelial cell adhesion on gelatin and albumin coatings to literature studies published so far for coating of polymeric substrates with these materials in general, inconsistent data can be found: gelatin-based coatings were described not to change [7, 9] or enhance endothelial cell adhesion compared to the uncoated polymeric substrate [4, 5, 37], but when compared to TCPS had lower cell numbers [4]. Albumin coatings showed similar endothelial adhesions compared to uncoated polytetrafluoroethylene [10], while cross-linked albumin-heparin gels were described to enhance endothelial cell adhesion and proliferation [38]. Howsoever, the high cell adhesion to gelatin-based coatings determined for microvascular cells in this study is a promising precondition for bio-integration of coated PET membranes in vivo.

The release of VEGF from the coatings was similar to previous studies from our group investigating macroscopic hydrogel samples: releases were dependent on the loading concentration [26] and possible interactions between the growth factor and the hydrogel matrix [25, 26]. Going a bit more into detail concerning the possible interaction between growth factor and hydrogels matrix, regular reports in literature show that the IEP affects the loading and release of growth factors [18–22]. In a previous study the GelA used in this study was found to have an IEP at pH 8.8, the GelB at pH 4.9, and GM5A5 at pH 4.5 [39]. Albumin was reported to have an IEP at pH 4.7 [17] and VEGF at pH 8.6 [40]. This means GelA and VEGF were charged positively at neutral pH, while GelB, albumin and GM5A5 were charged negatively. Polyion complexation which is often described to lower the releases was therefore not possible with VEGF and GelA, as determined in our previous study as well [25]. Polyion complexation is however possible with the other biopolymers, resulting in the observed lower initial and overall releases compared to GelA-Hep. The faster release of GM5A5-based compared to GelB-coatings on the other hand can probably be explained by a lower hydrophilicity of the GM5A5 hydrogels due to the inserted non-polar methacryl residues. In fact lower hydrodynamic radii of methacryl-modified gelatins were measured in aqueous solution compared to unmodified gelatin, and associated with lower hydrophilicity [39]. This change in chemical composition of the storage and release matrix was found to affect VEGF release out of GM/A hydrogels before [26].

Still, despite the different release profiles, GelA-Hep and GelB-Hep loaded with VEGF induced significant vessel formation in the CAM assay, while Alb-Hep coatings did not induce angiogenesis, although releasing comparable VEGF amounts as GelB-Hep. Therefore, finally the sustained release of VEGF did only lead to a pro-angiogenic response in combination with the observed advantageous properties of GelA-Hep and GelB-Hep for HDMVEC adhesion and elevated HDMVEC metabolic activity. GM5A5-HepM5 coatings showed favorable cell adhesion properties, but due to low VEGF release and possibly, also due to lower metabolic cell activity no pro-angiogenic response was induced in this case. This issue has so far to our knowledge not been described in literature, where usually VEGF-release is seen as the decisive factor for a pro-angiogenic response. Still, the data in this study suggest that also other factors have to be considered carefully i.e. endothelial cell adhesion and metabolic activity.

## Conclusions

We successfully prepared biopolymer coatings on PET membranes and studied their performance in terms of VEGF release, and support of endothelial cell functions. The results indicate that besides release properties for VEGF also additional aspects such as endothelial cell adhesion and metabolic activity of adhesive cells in vitro have to be taken into account to predict whether materials and coatings induce angiogenesis: The VEGF release from carbodiimid-cross-linked, negatively charged GelB-Hep coatings and Alb-Hep coatings was nearly identical, however, gelatin-heparin coatings showed favourable high HDMVEC adhesion, while albumin-heparin coatings did not change cell adhesion significantly compared to the uncoated PET membranes. Interestingly, the VEGF loaded Gel-Hep coatings (GelA-Hep and GelB-Hep) induced angiogenesis in the chorioallantois membrane assay *ex ovo*, but Alb-Hep coatings did not.

In general, VEGF release from the investigated thin biopolymer-hydrogel coatings was dependent on the IEP, composition, and loading concentration in the same way as described for bulk hydrogels with similar composition. The release from the radical cross-linked methacryl-modified gelatin GM5A5 with hydrophobic (poly) methacryl domains was significantly lower than the carbodiimid-cross-linked counterparts. All coatings prepared on porous PET foil in this study showed good coating homogeneities and stability over 4 weeks under cell culture conditions.

Taking all material properties into account, thin hydrogel coatings based on carbodiimide-cross-linked gelatins type A or type B with heparin are most promising for the preparation of pro-angiogenic coatings and should be further considered for in vivo studies.

## Supplementary information

Supplementary Figure S1

Supporting Information
