# Supplementary figures and images for: The choice of biopolymer is crucial to trigger angiogenesis with vascular endothelial growth factor releasing coatings
Source: J Mater Sci Mater Med. 2020 Oct 27;31(11):93. doi: 10.1007/s10856-020-06424-3 (PMC7591429; doi:10.1007/s10856-020-06424-3)

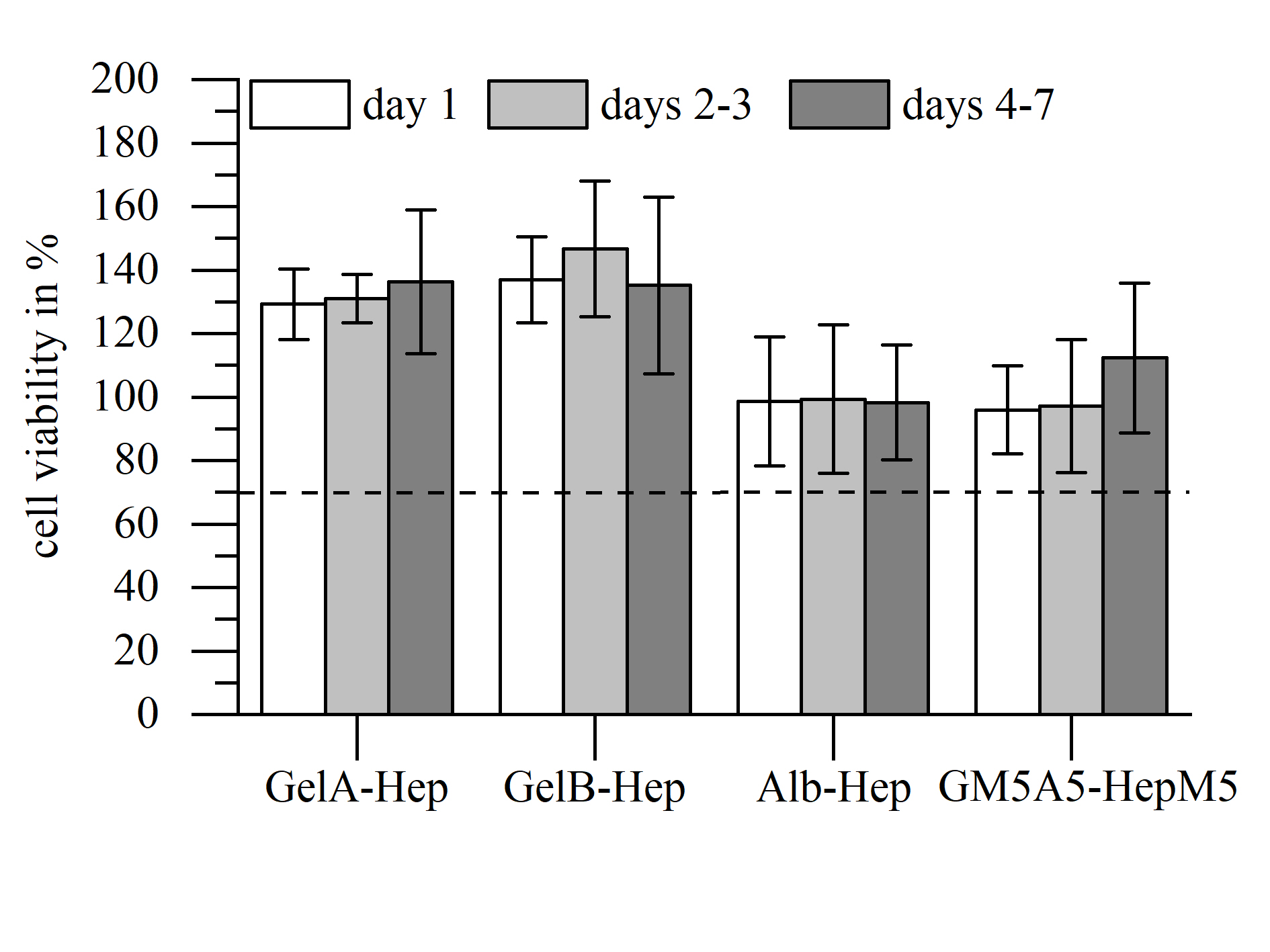

Supplement: Supplementary file 1 — Supplementary Figure S1 [file 10856_2020_6424_MOESM1_ESM.jpg]
