# Supplementary material for: The choice of biopolymer is crucial to trigger angiogenesis with vascular endothelial growth factor releasing coatings
Source: J Mater Sci Mater Med. 2020 Oct 27;31(11):93. doi: 10.1007/s10856-020-06424-3 (PMC7591429; doi:10.1007/s10856-020-06424-3)
Supplement: Supplementary file 2 — Supporting Information [file 10856_2020_6424_MOESM2_ESM.docx]

Supporting Information

The Choice of Biopolymer is Crucial to Trigger Angiogenesis with Vascular Endothelial Growth Factor Releasing Coatings

Christiane Claaßen, Miriam Dannecker, Jana Grübel, Maria-Elli Kotzampasi, Günter E. M. Tovar, Boris V. Stanzel, Kirsten Borchers

# Cytocompatibility

Cytocompatibility of hydrogel materials was evaluated via an MTS cell proliferation assay with human dermal microvascular endothelial cells (HDMVECs) according to DIN EN ISO 10993-12 and 10993-5. Macroscopic hydrogels were prepared analogous to the hydrogel pre-cursor solutions for membrane coating in 2.2 and 2.3, by pipetting this hydrogel-precursor solution in an aluminum mold and curing as described in the manuscript. Hydrogel cylinders of 8 mm diameter were punched out, washed for at least 24 h in PBS^+^ at 37 °C and dried in vacuum. Hydrogel samples (8 mm) were then incubated in ECGM (2 mL) for seven days at 37 °C under gentle shaking to prepare the hydrogel extracts. Medium was exchanged after 24 h, 3 d and 7 d, meaning that the whole supernatant was removed and replaced by new ECGM. ECGM was used as negative control (NC), ECGM supplemented with 1 % SDS was used as positive control (PC). Endothelial cells were isolated from human skin biopsies received from the Robert-Bosch-Krankenhaus, Klinik Charlottenhaus, Stuttgart (Germany) and cultivated as previously described in [1] and used in passage 3 for the experiments. After 24 h of incubation extracts were removed, cells were incubated with MTS solution (ECGM:MTS=5:1) for 3.5 h Absorbance of the formazan was measured with a fluorescence microwellplate reader (Tecan Reader Synergy 2) from BioTek (Germany) at 492 nm. The mean of the absorbance of the NCs was set to 100 % cell viability and all other measured data were normalized to it. Samples were considered to be non-toxic according to DIN EN ISO 10993-5 at viabilities higher than 70 % with respect to NC. The assay was conducted with cells of three healthy donors and hydrogels of respectively three independently prepared batches.


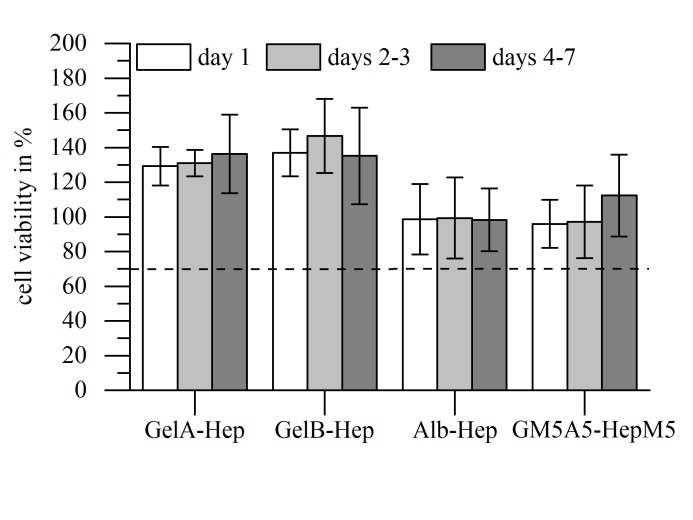


Figure S1 Compatibility of hydrogel extracts in endothelial cell growth medium with endothelial cells. GelA-Hep and GelB-Hep extracts resulted in higher cell viabilities compared to the viability (100 %) of control cells cultured in standard endothelial cell growth medium (100 %, *p* < 0.05), while Alb-Hep and GM5A5-HepM5 extracts showed similar viabilities to the control (*p* > 0.1). (*n*=3)

# XPS analysis of uncoated membrane

Table S1 Elemental composition derived from XPS analysis; values are given in atom%.

| Element | E_B_ in eV | atom% |  |
| --- | --- | --- | --- |
| C3 | 288.6 | 12.3 | **C**OOH [2] |
| C2 | 286.2 | 16.0 | **C**-O-C / **C**-OH [2] |
| C1 | 284.6 | 43.7 | **C**-C / **C**-H [2] |
| O4 | 535.0 | 0.5 | C-**O**H [3] |
| O3 | 533.2 | 12.8 | **O**-C=O-Phenyl [3, 4] |
| O2 | 531.6 | 13.1 | O-C=**O**-Phenyl [3, 4] |
| O1 | 530.1 | 0.3 | Ar-C**OO**H [4] |
| Na, Si, S, Cl |  | traces |  |

# IR spectra of coated and uncoated PET membranes


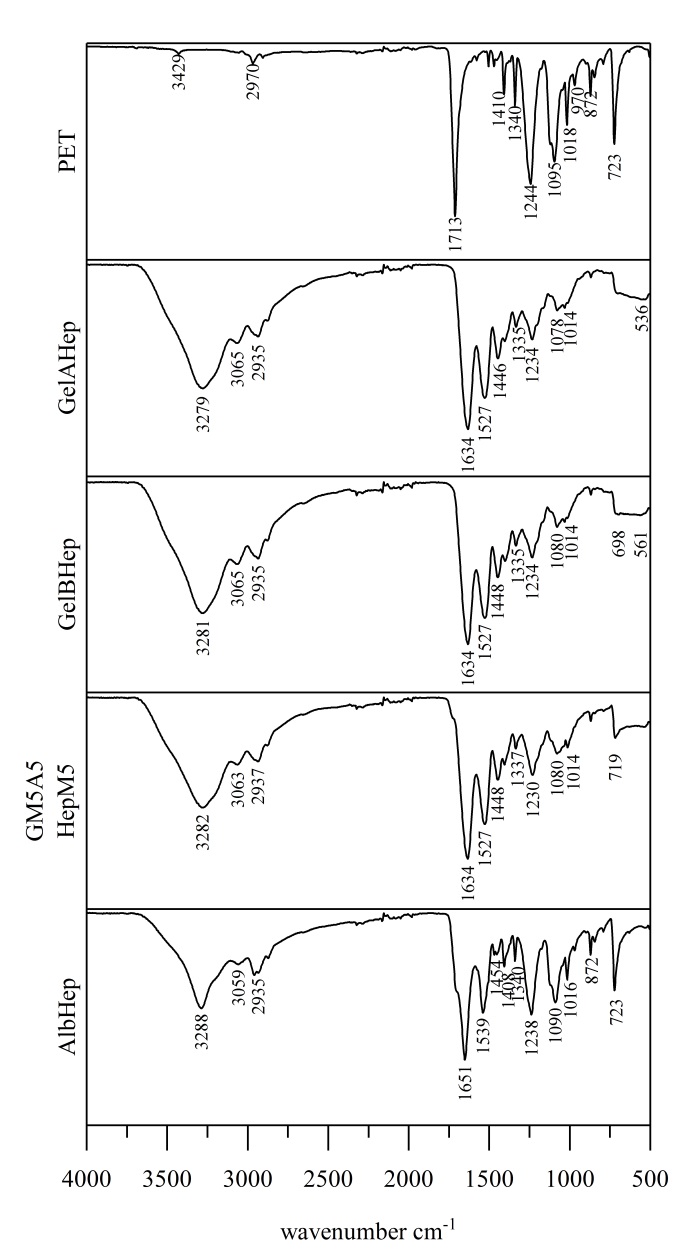


Figure S2 Infrared spectra of uncoated polyethylene terephthalate (PET) membrane and of biopolymer-coated PET membranes. PET: 3429 cm^-1^ OH, 1713 cm^-1^ C=O (COOH); biopolymer coatings ≈3280 cm^-1^ NH, ≈1630-1640 cm^-1^ C=O (CONHR).

# Quantification of Release of Vascular Endothelial Growth Factor

Table S2 Cumulated release of vascular endothelial growth factor in ng per membrane.

|  | GelA-Hep | | | | GelB-Hep | | | |
| --- | --- | --- | --- | --- | --- | --- | --- | --- |
| loading | 0.1 µg/mL | | 1.0 µg/mL | | 0.1 µg/mL | | 1.0 µg/mL | |
| release time  in days | average | ± | average | ± | average | ± | average | ± |
| 0.25 | 6.1 | 4.0 | 58.6 | 25.2 | 2.2 | 1.5 | 31.0 | 15.1 |
| 1 | 15.6 | 6.5 | 134.6 | 37.5 | 4.1 | 2.0 | 62.6 | 21.6 |
| 2 | 20.7 | 5.0 | 205.2 | 34.6 | 6.2 | 2.4 | 92.4 | 23.1 |
| 5 | 27.9 | 4.7 | 298.1 | 44.0 | 9.4 | 3.3 | 134.0 | 34.3 |
| 7 | 32.0 | 2.7 | 348.5 | 24.8 | 11.2 | 2.0 | 161.9 | 23.2 |
| 14 | 38.4 | 4.4 | 420.2 | 39.7 | 14.0 | 2.6 | 203.4 | 40.7 |
| 21 | 48.9 | 12.9 | 497.4 | 37.4 | 16.2 | 1.9 | 229.2 | 19.7 |
| 28 | 50.0 | 0.4 | 508.6 | 0.3 | 16.9 | 1.0 | 238.5 | 3.6 |
|  |  |  |  |  |  |  |  |  |
|  | Alb-Hep | | | | GM5A5-HepM5 | | | |
| loading | 0.1 µg/mL | | 1.0 µg/mL | | 0.1 µg/mL | | 1.0 µg/mL | |
| release time  in days | average | ± | average | ± | average | ± | average | ± |
| 0.25 | 3.7 | 3.2 | 75.8 | 13.5 | 1.2 | 0.2 | 16.4 | 6.8 |
| 1 | 9.4 | 3.0 | 117.3 | 6.7 | 1.8 | 0.1 | 40.3 | 7.4 |
| 2 | 11.2 | 1.2 | 150.6 | 6.8 | 2.2 | 0.0 | 50.8 | 2.3 |
| 5 | 13.4 | 1.3 | 182.4 | 8.9 | 2.5 | 0.1 | 56.9 | 0.8 |
| 7 | 14.0 | 0.4 | 198.4 | 5.2 | 2.7 | 0.1 | 59.6 | 0.2 |
| 14 | 14.6 | 0.4 | 222.3 | 4.1 | 2.8 | 0.0 | 61.8 | 0.5 |
| 21 | 14.8 | 0.2 | 233.4 | 1.5 | 2.9 | 0.0 | 63.6 | 0.3 |
| 28 | 15.5 | -- | 246.2 | 5.5 | 3.0 | 0.0 | 65.2 | 0.5 |

# Endothelial Cell Adhesion

#
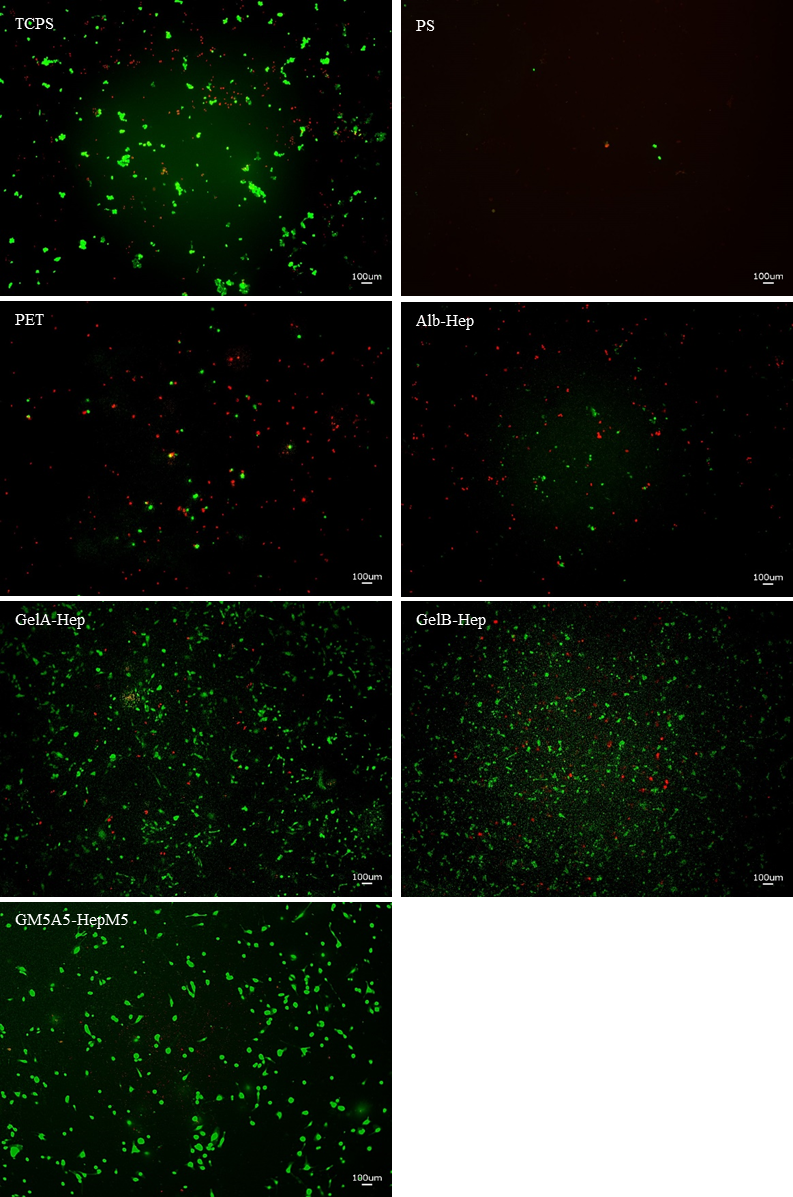


Figure S3 Adhesion of human dermal microvascular endothelial cells to tissue culture polystyrene (TCPS, positive control), polystyrene (PS, negative control), uncoated polyethylene terephthalate membrane (PET) and different biopolymer-based coatings.

# References

1. Volz A-C, Huber B, Schwandt AM, Kluger PJ. EGF and hydrocortisone as critical factors for the co-culture of adipogenic differentiated ASCs and endothelial cells. Differentiation. 2017;95:21-30.

2. Korolkov IV, Mashentseva AA, Güven O, Niyazova DT, Barsbay M, Zdorovets MV. The effect of oxidizing agents/systems on the properties of track-etched PET membranes. Polymer Degradation and Stability. 2014;107:150-7.

3. Cruz SA, Zanin M, Nascente PAP, Bica de Moraes MA. Superficial modification in recycled PET by plasma etching for food packaging. Journal of Applied Polymer Science. 2010;115(5):2728-33.

4. Beamson G, Briggs D. High Resolution XPS of Organic Polymers: The Scienta ESCA300 Database. Wiley; 1992.
